# Modelling population dynamics, health system, and unmet need toward health burden: scenarios in ASEAN and WHO SEARO countries

**DOI:** 10.7189/jogh.16.04194

**Published:** 2026-06-19

**Authors:** Kazi Fayzus Salahin, Tippawan Liabsuetrakul

**Affiliations:** 1 PSU Center for Global Health Research and Innovation (C-GHRi), Faculty of Medicine, Prince of Songkla University, Hat Yai, Songkhla, Thailand; 2 Department of Epidemiology, Faculty of Medicine, Prince of Songkla University, Hat Yai, Songkhla, Thailand

## Abstract

**Background:**

Universal health coverage is a cornerstone for achieving good health and well-being; however, its interaction with demographic and migration pressures remains underexplored, particularly in Southeast Asia. This study investigated how demographic pressure, migration pressure, health system capacity, and universal health coverage (UHC) influenced health burden across World Health Organization South-East Asia Region and Association of Southeast Asian Nations countries between 2000 and 2021 and simulated using a scenario-based model to forecast health burdens in 2030 and 2050.

**Methods:**

Multiple data sets obtained from Global Repositories, Burden of Disease, Global Health Observatory, World Bank Open Data portal, and International Organization for Migration were extracted. Missing data were addressed using multiple imputations. Composite domains were validated using principal component analysis. Pathways were estimated using multilevel structural equation modelling, and temporal effects were assessed using linear mixed-effects models. Sensitivity analyses were performed to test alternative UHC rollout periods.

**Results:**

Migration pressure was positively associated with health system capacity (β = 0.757) and UHC (β = 0.553), both *P* < 0.001. Health system capacity increased UHC (β = 0.582), while demographic pressure reduced it (β = −0.800). Health burden was inversely associated with UHC (β = −0.679) and migration pressure (β = −0.446). Mediation analysis confirmed indirect effects of UHC. Linear mixed-effects models showed that disability-adjusted life years declined before UHC rollout, increased immediately after rollout, then decreased with higher UHC coverage. Scenario-based forecasts indicate moderate reductions by 2030 and greater declines by 2050, particularly in countries with strong and well-structured health systems.

**Conclusions:**

UHC directly reduces health burden, whereas migration, health system capacity, and demographic pressure exert indirect effects. Forecasts indicate declining burden by 2030 and 2050, particularly in countries with strong health care systems. Continuous monitoring of sociodemographic pressures – along with sustained system capacity and UHC expansion – is essential for long-term health improvements in Southeast Asia.

The global health burden landscape is increasingly defined as a ‘poly-crisis’, in which demographic, environmental, and epidemiological shifts have converged to place unprecedented strain on health systems [1–3]. Health burden is commonly measured using disability-adjusted life years (DALYs). Globally, the burden has shifted from being dominated by premature mortality to a greater contribution from years lived with disability, reflecting the profound effects of population ageing and epidemiological transition [4,5]. This shift highlights how longer life expectancy, changing population structures, and uneven health system capacity are reshaping the balance between mortality and disability. DALYs provide a critical lens for understanding how demographic and system pressures interact to shape health burden at national and regional levels [6,7].

South and Southeast Asia have not been exempt from these evolving health burden patterns [8,9]. The Association of Southeast Asian Nations (ASEAN) and the World Health Organization (WHO) South-East Asia Region (SEARO) are undergoing profound demographic and epidemiological transitions that are reshaping health system demands and highlighting gaps in system preparedness [10–12]. The region is home to over 2.1 billion people, representing – approximately 25% of the global population [13], and is characterised by substantial social, economic, and political diversity, which contributes to pronounced health disparities [14]. Key demographic pressures include rapid population aging and urbanisation. Between 2000 and 2023, the population aged ≥65 years in Southeast Asia increased by 60%, with Thailand, Sri Lanka, and Indonesia leading the shift toward aging societies [15]. Urbanisation has accelerated, with over 50% of the region’s population now residing in urban areas, often in informal settlements with limited access to care [16,17]. Collectively, these trends place mounting pressure on health care systems that are already confronting a triple burden of diseases, including communicable diseases, maternal mortality, and undernutrition [18]. Migration pressure further accelerates this burden. Globally, migrants and refugees face up to a 30% higher risk of delayed care and unmet health needs compared with host populations [19]. Only a few member states – including Timor-Leste, Maldives, and Thailand – have made notable progress in integrating migrant health into their national frameworks [20], by establishing formal migrant health policies and dedicated budgeted implementation plans [21]. This contributes to uneven health-system capacity across the region.

In this region, health workforce shortages continue to constrain the delivery of efficient health care, with low health workforce density and limited hospital bed availability remaining well below Organisation for Economic Co-operation and Development (OECD) standards [17]. Policy inclusion – particularly the extent to which national health strategies address the needs of migrants and other vulnerable groups – remains unclear. Progress towards universal health coverage (UHC) has advanced regionally, with the service coverage index, a composite measure of essential health services, increasing from 47 in 2010 to 62 in 2021 [22,23]. However, the gains have been uneven because of health workforce shortages, financing constraints, and gaps in migrant coverage. Consequently, countries such as Myanmar and Timor-Leste lag behind better-performing systems in Thailand, Singapore, and the Maldives [9].

This limited institutional commitment reveals a critical policy gap across the region. Despite growing evidence of population aging, migration pressures, and rising health burdens across ASEAN and WHO SEARO countries, existing studies have tended to examine these factors in isolation. Few frameworks holistically integrate population dynamics, migration pressures, health system capacity, and policy inclusion to explain unmet health needs, particularly in maternal care, chronic disease management, infectious disease control, and migrant health services, which contribute to trends in DALYs across countries [9,24–27]. Although similar analyses have been conducted in the African region [28,29], Southeast Asia still lacks comprehensive studies that jointly examine UHC expansion, migration, and demographic pressures. To address this gap, this study aimed to investigate how demographic pressure, migration pressure, health system capacity, and UHC have influenced health burden across SEARO and ASEAN countries between 2000 and 2021 and simulate a scenario-based model to forecast the health burdens in 2030 and 2050. This study provides a reproducible, data-driven framework for understanding and forecasting the health burdens in these regions.

## METHODS

### Study design and settings

This study employed a longitudinal ecological design, in which aggregated country-level data were analysed to examine associations between population-level factors and health outcomes across 18 countries including 10 countries in WHO SEARO and 11 countries in ASEAN. The countries included were Bangladesh, Bhutan, Brunei Darussalam, Cambodia, the Democratic People’s Republic of Korea, India, Indonesia, the Lao People’s Democratic Republic, Malaysia, Maldives, Myanmar, Nepal, Philippines, Singapore, Sri Lanka, Thailand, Timor-Leste, and Vietnam, while Thailand, Timor-Leste, and Myanmar are unique in being members of both SEARO and ASEAN. The analytical period spanned from 2000 to 2021, enabling the assessment of temporal trends before and after the official UHC rollout.

### Data sources

This study used secondary data from multiple global repositories including the Global Health Data Exchange, Global Burden of Disease Study, WHO Global Health Observatory, World Bank Open Data portal, and the International Organization for Migration, covering the period 2000 to 2021 across countries in SEARO and ASEAN. Data were presented at selected time points (2000, 2005, 2010, 2015, 2017, 2019, and 2021), consistent with the UHC reporting pattern. Prior to UHC rollout, reporting occurred at 5-year intervals, whereas following its implementation, data were updated every two years to reflect more frequent monitoring. DALYs were obtained from the Global Burden of Diseases Study 2023 release, which provides estimates up to 2021, via the Global Health Data Exchange [30]. The UHC service coverage index and health system indicators were retrieved from the WHO Global Health Observatory using country-level filter [31]. Demographic indicators including population growth rate, dependency ratio, proportion aged 65+, and proportion of urban population, and economic indicators comprising gross domestic product (GDP) per capita, population size, and life expectancy, were sourced from the World Bank Open Data portal [32]. Migration statistics including net migration rate per 1000 population, share of international migrants in the total population, and remittance inflows as a proportion of GDP were collected from the International Organization for Migration (IOM) [33,34]. The final data set was structured as a country-year panel, comprising approximately 126 observations. The total number of observations was approximate due to missing data for some countries and years.

### Variables and their definitions

**Figure 1:**
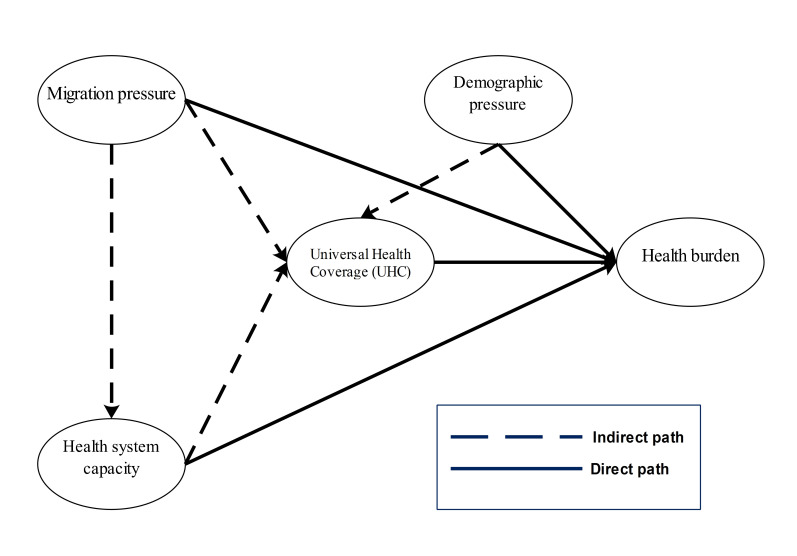
Conceptual framework of model analyses.

The primary outcome was the health burden index, measured by DALYs per 100 000 people, a standard composite measure of health burden [5]. Four composite domains were constructed by combining multiple indicators consistent with prior literature. The demographic pressure domain included a composite of population growth, dependency ratio, proportion aged 65+, and urbanisation [35,36]. The migration pressure domain included a composite of net migration, migrant stock, and remittance inflow [37,38]. The health system capacity domain included a composite of health workforce density per 10 000 population, hospital bed availability per 1000, and health expenditure [39,40]. The UHC domain was represented by service coverage proportion [41]. All variables were standardised using z-scores. Based on that we developed the relationship and presented in **Figure 1****.**

In this analysis, ‘UHC rollout year’ was defined as the first year in which each country formally adopted a national policy or programme aimed at achieving universal health coverage, as presented in Table S1 in the **Online Supplementary Document** [23,42–44]. Standard definitions of all included variables are provided in Table S2 in the **Online Supplementary Document** [45–56]. The composite domains – migration pressure, universal health coverage (UHC), demographic pressure, and health system capacity – were validated using principal component analysis (PCA) to assess whether the selected indicators adequately represented each domain as presented in Table S3 in the **Online Supplementary Document****.**

### Data management and harmonisation

Data cleaning and harmonisation involved replacing placeholder strings with missing values, converting numeric variables into appropriate formats, factorising categorical identifiers such as country codes, and standardising temporal variables to an integer year format for time-series modelling. Missingness was assessed across all variables, and moderate gaps anticipated in remittance inflow, hospital beds per 1000 population, and health workforce density were addressed using multiple imputations with predictive mean matching to preserve distributional characteristics. To ensure transparency, missing data were imputed and flagged using a separate indicator variable. All numerical variables were standardised using z-scores. Composite indices were constructed by averaging standardised indicators within each domain. PCA was used to assess whether the selected indicators adequately represented each domain.

### Statistical analysis

A combination of statistical approaches was used, including linear mixed-effects models (LMMs) estimated using restricted maximum likelihood. These models were used to estimate population-level fixed effects, average effects across all countries, and country-specific random effects indicating deviations for individual countries, before and after the UHC rollout. Best Linear Unbiased Predictors (BLUPs) were extracted to characterise country-specific deviations and temporal trends following UHC implementation.

Scenario-based forecasting was anchored to 2021, with forecast horizons set to 2030 and 2050. We selected 2021 as an anchored point, as it represents the most recent year with complete data. The years 2030 and 2050 were selected as future reference points, corresponding to the United Nations Sustainable Development Goals target year and long-term demographic and health system planning horizons, respectively. Three scenarios were defined:

(1) a baseline scenario, applying an average yearly change to all predictors;

(2) an optimistic scenario, estimating maximal observed improvements in UHC and system capacity alongside minimum migration and demographic pressures;

and (3) a pessimistic scenario, assuming UHC progress stalled at 2021 levels, combined with maximum migration and demographic pressures and minimum system capacity.

The predicted health burden indices under each scenario were derived from the fitted LMM using the observed annual changes in the predictors.

In this analysis, positive coefficients were interpreted as risk effects, indicating an association between the predictor and increased health burden. In contrast, negative coefficients were interpreted as protective effects, indicating an association between the predictor and reduced health burden. Both unstandardised and standardised coefficients were reported. Unstandardised coefficients (β) represent the change in the outcome associated with a one-unit increase in the predictor, whereas standardised coefficients (std. β) reflect the relative strength of associations in standard deviation units.

## RESULTS

The key indicators of each latent domain of demographic pressure, migration pressure, health system capacity, UHC, health burden by selected time points and overall average are descriptively presented in Table S4 in the **Online Supplementary Document**. Demographic pressure, UHC service coverage, and DALYs were consistently reported across all countries and years (n = 126). In contrast, migration pressure showed substantial missingness (n = 70), particularly the indicators of international migrant stock after 2015 and remittance inflows in 2021. Indicators of health workforce density and hospital bed availability in the health system capacity domain were not reported in approximately 10% of countries prior to 2010.

A multilevel structural equation model of the pathways linking the four domains and health burden, based on the conceptual framework, is shown in **Table 1**. Migration pressure was positively associated with health system capacity (β = 0.757, *P* < 0.001) and UHC expansion (β = 0.553, *P* < 0.001). Health system capacity also predicted positive progress towards UHC (β = 0.582, *P* < 0.001), while demographic pressure exerted a negative effect on UHC (β = −0.800, *P* < 0.001). For health burden outcomes, UHC exerted a significant negative effect (β = −0.679, *P* < 0.001), and migration pressure also directly reduced burden (β = −0.446, *P* < 0.001). The direct effects of health system capacity and demographic pressure on health burden were not significant.

**Table 1:** Multilevel structural equation model of pathways linking four domains and health burden based on conceptual framework

Path	Estimate β	Std. Error	z-value	*P*-value*	Std. β	Mediation Type
**Direct effects**						
Health system capacity ← Migration pressure	0.757	0.120	6.289	<0.001	0.489	
UHC ← Migration pressure	0.553	0.171	3.243	<0.001	0.286	
UHC ← Health system capacity	0.582	0.101	5.756	<0.001	0.465	
UHC ← Demographic pressure	−0.800	0.249	−3.213	<0.001	−0.253	
Health burden ← UHC	−0.679	0.057	−11.825	<0.001	−0.683	
Health burden ← Migration pressure	−0.446	0.115	−3.897	<0.001	−0.232	
Health burden ← Health system capacity	−0.117	0.073	−1.598	0.110	−0.094	
Health burden ← Demographic pressure	0.220	0.167	1.316	0.188	0.070	
**Indirect effects (mediation)**						
Migration pressure → UHC → Health burden	−0.376	0.120	−3.127	0.002	−0.195	Partial
Health system capacity → UHC → Health burden	−0.395	0.076	−5.176	<0.001	−0.317	Partial
Demographic pressure → UHC → Health burden	0.544	0.175	3.101	0.002	0.173	Partial
Migration pressure → Health system capacity → Health burden	−0.089	0.057	−1.549	0.121	−0.046	No mediation
**Total effects**						
Migration pressure → Health burden	−1.210	0.156	−7.774	<0.001	−0.628	
Health system capacity → Health burden	−0.512	0.095	−5.411	<0.001	−0.411	
Demographic pressure → Health burden	0.763	0.233	3.273	0.001	0.243	

Std. β – standardised coefficients, UHC – universal health coverage

*Statistical significance was defined as *P* < 0.05.

Mediation analyses confirmed that the effects of migration pressure (β = −0.376, *P* = 0.002), health system capacity (β = −0.395, *P* < 0.001), and demographic pressure (β = 0.544, *P* = 0.002) on health burden were partially mediated by UHC, accounting for approximately 31, 77, and 71% of the respective total effects. Total effects highlighted migration pressure (β = −1.21, *P* < 0.001) and health system capacity (β = −0.51, *P* < 0.001) as major protective factors, largely mediated by improvements in UHC, while demographic pressure increased overall health burden (β = 0.76, *P* = 0.001).

Linear mixed-effects model examining the health burden index in relation to UHC progress across SEARO and ASEAN countries is presented in **Table 2**. At the population level (Panel A), the pre-UHC trend showed a significant negative slope (β = −0.074, *P* < 0.001), indicating that DALYs were declining prior to UHC rollout. The immediate effect of UHC rollout was positive (β = 0.128, 95% CI = 0.008, 0.248), suggesting a short-term increase in measured burden, likely reflecting improved access to care and improved detection. However, the wide confidence interval indicated that this estimate should be interpreted with caution. The post-UHC trend was strongly positive (β = 0.093, 95% CI = 0.063, 0.123), indicating that DALYs constantly increased over time. The UHC index exhibited a significant negative slope (β = −0.185, 95% CI = −0.300, −0.070), demonstrating that higher levels of UHC coverage were associated with reduced health burden. Other covariates showed non-significant effects at the population level. Random effects (Panel B) highlighted substantial heterogeneity across countries. Negative UHC index values were observed in Nepal, followed by Bangladesh, Timor-Leste, Lao PDR, and Cambodia, all of which showed negative pre-UHC trends and substantial UHC rollouts. The highest positive UHC index value was observed in Singapore, with similar patterns in Brunei Darussalam, Malaysia, and Thailand, reflecting smaller incremental gains from UHC expansion in countries with strong baseline systems and no adverse outcomes.

**Table 2:** Fixed and random effects from the linear mixed-effects model of health burden index by UHC progress across SEARO and ASEAN countries

Panel A. Population-level fixed effects: Health burden									
**Predictor**				**Estimate**		**95% CI**		*P*-value*	
(Intercept)				−0.175		−0.469, 0.120		0.261	
Time (pre-UHC trend)				−0.074		−0.102, −0.047		<0.001	
UHC rollout (immediate level change)				0.128		0.008, 0.248		0.052	
Time post-UHC (post-UHC trend)				0.093		0.063, 0.123		<0.001	
Demographic pressure index				0.093		−0.211, 0.397		0.552	
Migration pressure index				-0.001		−0.180, 0.178		0.989	
Health system capacity index				0.101		−0.031, 0.232		0.137	
UHC index				-0.185		−0.300, −0.070		0.002	
**†Panel B. Country-level random effects (best linear unbiased predictors): Health burden**									
**Country**	**Time (pre-UHC trend)**	**UHC Rollout (Immediate level change)**	**Time post-UHC (post-UHC trend)**		**Demographic pressure**		**Migration pressure**	**Health system capacity**	**UHC index**
Bangladesh	−0.105	0.204	0.122		−0.201		−0.351	−0.589	−0.895
Bhutan	−0.147	0.323	0.124		−0.207		−0.147	−0.512	−0.331
Brunei Darussalam	−0.060	0.085	0.054		−0.003		0.449	0.514	0.976
Cambodia	−0.114	0.214	0.099		−0.185		−0.297	−0.637	−0.349
Dem. People’s Rep. of Korea	−0.083	0.119	0.077		0.41		−0.308	2.012	0.197
India	−0.058	0.094	0.125		−0.156		−0.284	−0.272	−0.223
Indonesia	−0.036	0.039	0.089		−0.017		−0.421	−0.561	−0.554
Lao People’s Dem. Republic	−0.126	0.261	0.157		−0.028		−0.563	−0.579	−0.742
Malaysia	−0.063	0.094	0.064		0.338		0.166	0.142	0.976
Maldives	−0.059	0.079	0.051		−0.012		0.59	0.765	0.233
Myanmar	−0.088	0.171	0.167		−0.386		−0.445	−0.483	−0.545
Nepal	−0.075	0.126	0.099		−0.178		0.525	−0.629	−0.930
Philippines	0.016	−0.095	0.103		0.055		0.08	−0.37	−0.223
Singapore	−0.039	0.024	0.018		0.46		1.308	1.558	1.665
Sri Lanka	−0.103	0.199	0.078		−0.218		−0.053	−0.068	0.215
Thailand	−0.111	0.222	0.091		−0.057		−0.269	−0.349	0.976
Timor-Leste	−0.065	0.114	0.105		0.614		0.206	0.291	−0.742
Vietnam	−0.023	0.027	0.050		−0.23		−0.185	−0.232	0.296

CI – confidence interval, WHO SEARO – World Health Organization South-East Asia Region, ASEAN – Association of Southeast Asian Nations, UHC – universal health coverage.

*Statistical significance was defined as *P* < 0.05.

†In Panel B, negative random effects indicate stronger-than-average protective associations of UHC or system capacity with reduced health burden, while positive values indicate weaker marginal effects relative to the regional average. Positive coefficients in countries with strong baseline systems reflect smaller incremental gains from UHC expansion but not adverse outcomes.

**Figure 2:**
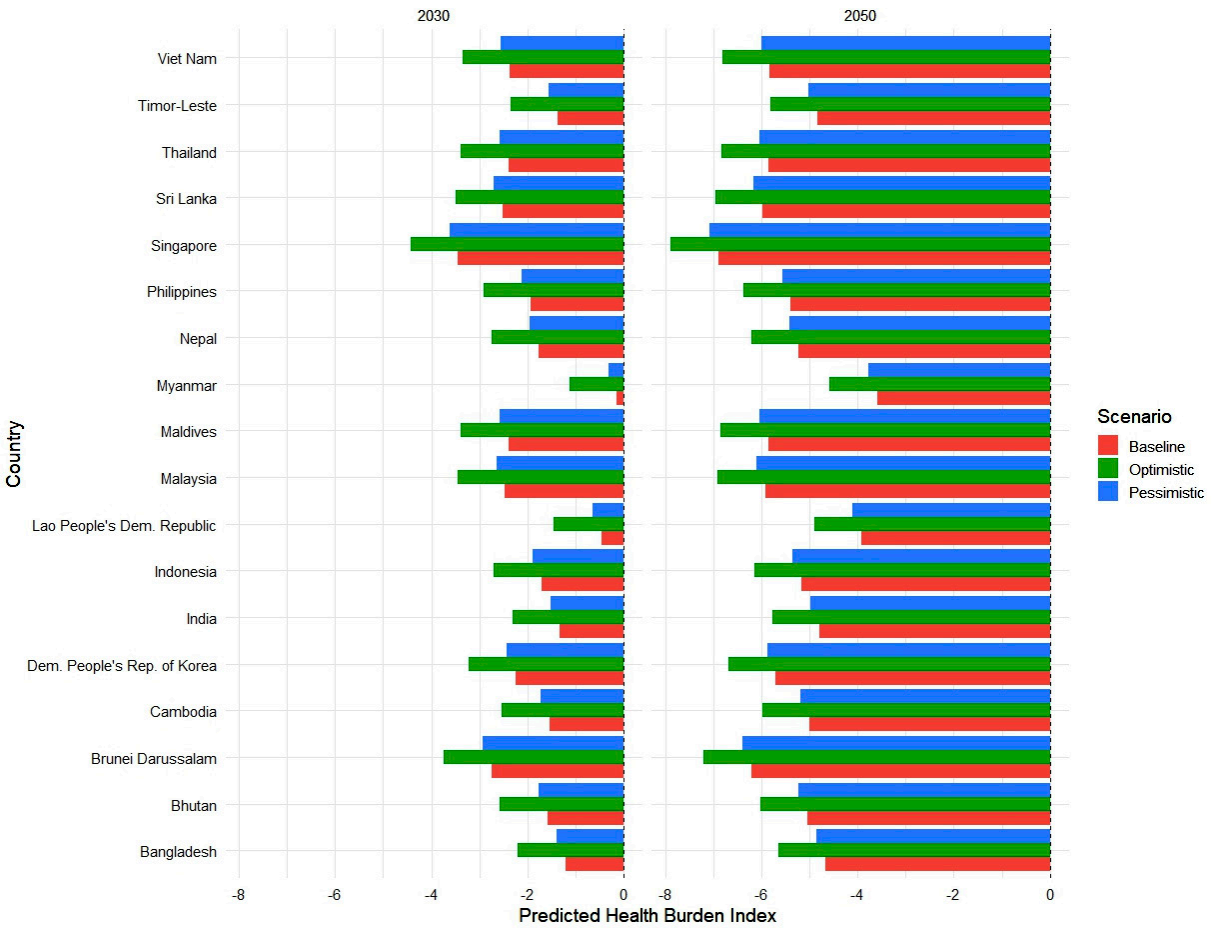
Scenario-based forecasts of health burden under baseline, optimistic, and pessimistic assumptions for the years 2030 and 2050.

The estimated health burden values for each scenario were derived from the observed year-period changes based on the four composite indicators presented in **Table 3**. The baseline average change in the health burden estimated for the four composite indicators was negative only for demographic pressure. The scenario-based forecasts of the health burden for 2030 and 2050 are shown in **Figure 2****.** Anchored in 2021, the baseline scenario forecasted a consistent reduction in health burden across all countries; however, the magnitude varied. By 2030, health burden declines were moderate across countries, typically between −0.5 and −2.0 units, and these reductions persisted through 2050. The optimistic scenario predicted the largest protective effects in 2030. Singapore and Brunei Darussalam showed reductions of approximately −5.0 and −4.0 units, respectively, expanding to approximately −8.0 and −7.0, respectively, in 2050. In contrast, Myanmar and Lao People’s Democratic Republic showed values close to −1.0 in 2030 and approximately −5.0 in 2050. Under the pessimistic scenario, reductions persisted, though smaller in magnitude with declines of approximately −1.0 to −1.5 units in Myanmar and Lao People’s Democratic Republic in 2030 and approximately −4.0 units in 2050, indicating that even limited improvements in UHC and system capacity can compound over time.

**Table 3:** Estimates of health burden scenarios using four composite indicators derived from a mixed-effects panel regression model (base year 2021)*

Domain	Pessimistic estimate (minimum change)	Baseline (average change)	Optimistic estimate (maximum change)
UHC index	1.880	0.287	−0.626
Migration pressure	0.844	0.017	−2.310
Health system capacity	1.550	0.055	−1.460
Demographic pressure	0.486	−0.034	−1.360

UHC – universal health coverage

*Scenario values are derived from observed year-period changes in the composite indicators. The mean changes define the baseline trajectories, whereas the minimum and maximum observed changes define the pessimistic and optimistic scenarios. Negative values indicate protective effects, and positive values indicate an increased health burden.

## DISCUSSION

Analysis across SEARO and ASEAN countries (2000–2021) showed that migration pressure was positively associated with both health system capacity and UHC, contributing directly and indirectly to a reduced health burden. In contrast, demographic pressure weakened UHC and increased the health burden. Health system capacity reduced the burden, partly mediated by UHC. Although DALYs were declining prior to UHC rollout, a short-term increase occurred, likely reflecting improved detection and access to care. Over time, higher UHC coverage consistently reduced the burden, with stronger effects observed in countries with weaker baseline systems and smaller gains in countries with stronger baseline systems. The projections for 2030 and 2050 indicated sustained reductions across scenarios, highlighting the long-term benefits of UHC expansion and health system strengthening.

Our analysis demonstrates that migration pressure is strongly associated with health system capacity and indirectly reduces health burden through UHC. This finding aligns with previous evidence suggesting that inclusive migration and displacement policies can accelerate UHC reform, thereby reframing migrants as a catalyst for strengthening the health system, rather than a strain on it [57,58]. This finding is also supported by a regional analysis of the Asia-Pacific health system, which demonstrated that migration pressure can indirectly benefit host populations [59], while country-level experiences – such as Thailand’s extension of UHC to migrant populations – demonstrates strengthened health system capacity and improved health outcomes [60].

Some studies have reported that migration increases the risk of overcrowded facilities, inequitable access, and financial constraints [33,61], which contrasts with our findings. This discrepancy may stem from our focus on migration pressures in relation to system adaptation and UHC, highlighting the potential long-term protective effects that may not be captured in exclusionary policy contexts, such as restrictive migration or health care access policies that limit coverage for certain groups. This suggests that the impact of migration is conditional; it can be protective when systems adapt inclusively but harmful under restrictive frameworks. In contrast, demographic pressure exerted a negative influence, weakening UHC and increasing the health burden. Our analysis shows that aging and urbanisation increase DALYs by increasing the proportion of years lived with disability, echoing global evidence of the epidemiological transition from mortality-dominated to disability-dominated DALYs [6,9]. This underscores the urgent need for health system preparedness for aging societies across Southeast Asia [62,63].

Health system capacity reduces the health burden, with part of this effect mediated by UHC [64]. Thailand provides a clear example: long-term investments in district-level infrastructure, rural health centres, and workforce distribution improved access, but substantial reductions in DALYs occurred only after the 2002 UHC rollout [65]. This pathway highlights UHC not only as a policy goal but also as a key mechanism through which system investments translate into measurable health gains [26]. UHC thus emerges as a cornerstone of resilience. Although DALYs were declining prior to the UHC rollout, a short-term increase followed, likely reflecting improved detection and access to care. Over time, higher UHC coverage consistently reduced the health burden [9,64,66]. Our results highlight the importance of distinguishing between short- and long-term protective effects.

Our scenario, anchored in 2021, forecasts consistent reductions in the health burden across all trajectories, consistent with previous research [59]. However, this differs from some global models [67] that project rising burdens on aging societies despite UHC expansion. This discrepancy likely reflects the fact that our regional model captures adaptive dynamics not visible in the global averages, underscoring the value of the regional analysis. Previous studies have typically examined individual components in isolation, with limited integration into a unified framework. For example, some analyses of UHC progress in the SEARO region have focused narrowly on workforce shortages without linking coverage expansion to the overall health burden [9]. Similarly, research on migration dynamics has described regional flows but has not connected them to DALY outcomes [25]. Other studies have highlighted the role of UHC in promoting equity but have not modelled interactions with demographic or migration pressures [26]. Similarly, studies on migrant inclusion in ASEAN health systems have documented policy gaps without assessing the mediating effects of UHC [68]. By integrating migration, demographic pressure, health system capacity, and UHC into a single framework, our study provides novel, quantitative insights into the drivers of health burden across SEARO and ASEAN countries.

We used principal component analysis to validate the theoretical construction of the composite indices and applied multiple complementary approaches to ensure methodological consistency. Scenario forecasts for 2030 and 2050 were then modelled to provide prospective insights into long-term trajectories under varying assumptions. We acknowledged several limitations. First, missing data were common in existing databases, particularly for migration and health system indicators; therefore, we applied multiple imputations with predictive mean matching to preserve distributional characteristics. Second, ecological aggregation may obscure within-country heterogeneity; however, we used standardised z-scores and composite indices to reduce measurement errors and improve comparability across countries. Third, exposure misclassification may arise from defining UHC rollout by formal adoption year; therefore, we conducted sensitivity analyses using recoded rollout definitions, yielding consistent results. Finally, although causal inference is limited by the ecological design, we triangulated direct, mediated, and total effects to strengthen confidence in the observed associations.

## CONCLUSIONS

Comprehensive UHC reduces health burden, both directly and indirectly, by mediating the effects of migration, health system capacity, and demographic pressure. Policymakers should interpret short-term increases in burden following UHC rollout as transitional effects driven by improved detection and access, while sustaining investments in system capacity and UHC expansion to achieve long-term reductions in the health burden. Future research should integrate household-level data with system indicators and extend forecasts to incorporate governance and financing dynamics, providing a better understanding of the contextual drivers of UHC effectiveness.

## Additional material


Online Supplementary Document
