# Supplementary material for: Modelling population dynamics, health system, and unmet need toward health burden: scenarios in ASEAN and WHO SEARO countries
Source: J Glob Health. 2026 Jun 19;16:04194. doi: 10.7189/jogh.16.04194 (PMC13334566; doi:10.7189/jogh.16.04194)
Supplement: Online Supplementary Document [file jogh-16-04194-s001.pdf]

Table S1. UHC rollout years for first year in which each country formally adopted a national policy or programme aimed at UHC

Country	UHC rollout year
Bangladesh	2012
Bhutan	2009
Brunei Darussalam	2000
Cambodia	2016
Democratic People’s Republic of Korea	2005
India	2018
Indonesia	2014
Lao People’s Democratic Republic	2016
Malaysia	2000
Maldives	2011
Myanmar	2017
Nepal	2015
Philippines	2019
Singapore	2000
Sri Lanka	2000
Thailand	2002
Timor-Leste	2015
Vietnam	2015

Abbreviation: UHC, universal health coverage.

Table S2. Definition of the variables[45-56]

Indicator	Definition
DALYs per 100,000	*"DALY is an abbreviation for disability-adjusted life year. It is a universal metric that allows researchers and policymakers to compare very different populations and health conditions across time. DALYs equal the sum of years of life lost (YLLs) and years lived with disability (YLDs). One DALY equals one lost year of healthy life. DALYs allow us to estimate the total number of years lost due to specific causes and risk factors at the country, regional, and global levels."*[45]
UHC Service coverage index (0–100)	*"The Universal Health Coverage (UHC) Service Coverage Index is a composite measure of access to essential health services. It is calculated as the geometric mean of 14 tracer indicators covering reproductive, maternal, newborn and child health, infectious diseases, non-communicable diseases, and service capacity/access. Reported on a scale from 0 (worst) to 100 (best)."*[46]
Health workforce density	*"Number of health workers (doctors, nurses, midwives, pharmacists, dentists) per 10,000 population, classified using ISCO-08 occupational codes."*[47]
Hospital beds per 1,000	*"Number of hospital beds available per 1,000 population, reported annually by WHO member states."*[48]
Health expenditure per capita	*"Per capita current health expenditure expressed in respective currency - US dollar."*[49]
Population growth rate (%)	*"Annual percentage increase in total population."*[50]
Dependency ratio (%)	*"Ratio of dependents (ages <15 and >64) to working-age population (15–64). Formula: 100 × (Population 0–14 + Population 65+) ÷ Population 15–64."*[51]
% aged 65+	*"Share of population total population 65 years of age or older. Population is based on the de facto definition of population, which counts all residents regardless of legal status or citizenship."*[52]
% urban population	*"Share of population living in urban areas, as defined by national statistical offices."*[53]

Net migration rate	*"Net migration is the net total of migrants during the period, that is, the number of immigrants minus the number of emigrants, including both citizens and noncitizens."*[54]
Migrant stock (% of population)	*"International migrant stock (% of population) is the proportion of people at mid-year born in a country other than that in which they live. It also includes refugees."*[55]
Remittance inflow (% GDP)	*"Workers' remittances and compensation of employees comprise current transfers by migrant workers and wages and salaries earned by nonresident workers. Data are the sum of three items defined in the fifth edition of the IMF's Balance of Payments Manual: workers' remittances, compensation of employees, and migrants' transfers."*[56]

Table S3. Composite domain validation using principal component analysis (PCA)

Domain	Variance Explained (Component 1)	Indicator	Key Loadings (Dimension 1)
Demographic pressure	55.7%	Rate of population change	-0.62
		Total dependency ratio	-0.85
		Population aged 65+	0.81
		Urban population %	0.69
Migration pressure	52.8%	Net migration rate (per 1,000)	0.78
		International migrant stock (% of population)	0.70
		Remittance inflow (% of GDP)	-0.69
Health system capacity	69.3%	Medical doctor density (per 10,000)	0.95
		Hospital beds per 1,000 population	0.76
		Health expenditure per capita (USD)	0.78
UHC index	-	UHC service coverage index (single indicator; no PCA conducted)	N/A

Abbreviations: UHC, universal health coverage; GDP, global domestic product; N/A, not applicable.

Table S4. Descriptive statistics of indicators for each latent domains of interest before imputation by years§

Indicator	Overall N = 126	2000 N = 18	2005 N = 18	2010 N = 18	2015 N = 18	2017 N = 18	2019 N = 18	2021 N = 18
**Demographic pressure**	**N = 126**							
Rate of population change (%)	1.20 [0.75, 1.66]	1.71 [1.41, 2.20]	1.48 [0.91, 1.66]	1.35 [0.78, 1.88]	1.20 [0.83, 1.45]	1.05 [0.75, 1.30]	0.96 [0.69, 1.51]	0.77 [0.63, 1.35]
Total dependency ratio	51 [44, 60]	64 [50, 78]	58 [48, 68]	51 [44, 62]	49 [44, 57]	48 [43, 57]	47 [43, 56]	47 [41, 54]
Population aged 65+ (%)	5.55 [4.21, 6.55]	3.85 [3.37, 5.98]	4.63 [3.41, 6.20]	4.94 [4.21, 6.05]	5.56 [4.57, 6.45]	5.83 [4.91, 6.77]	6.14 [5.29, 7.25]	6.34 [5.59, 7.89]
Urban population (%)	36 [28, 53]	27 [24, 46]	30 [27, 46]	33 [29, 50]	36 [30, 53]	38 [30, 55]	39 [31, 56]	40 [32, 57]
**Migration pressure**	**N = 70**							
Net migration rate (per 1,000)	0 [-2, 1]	0 [-2, 5]	-1 [-4, 2]	0 [-3, 1]	0 [-2, 1]	0 [-2, 1]	0 [-1, 1]	0 [-1, 0]
International migrant stock (% of population)	1 [0, 7]	1 [0, 5]	1 [0, 6]	1 [1, 7]	1 [0, 7]	NA	NA	NA
Remittance inflow (% of GDP)	2.0 [0.5, 5.6]	1.8 [0.7, 3.7]	1.2 [0.2, 5.5]	1.3 [0.5, 7.3]	3.1 [1.0, 6.6]	2.6 [0.9, 5.4]	2.9 [1.0, 5.2]	NA
**Health system capacity**	**N = 87**							
Medical doctors' density (per 10,000)	7 [4, 12]	7 [3, 12]	6 [4, 11]	5 [4, 12]	6 [4, 8]	8 [5, 17]	8 [5, 10]	8 [6, 15]
Hospital beds per 1,000 population	1.70 [1.01, 2.39]	1.70 [1.09, 2.15]	1.58 [0.66, 2.29]	1.82 [0.86, 2.15]	1.71 [1.01, 2.44]	1.69 [1.05, 2.55]	1.82 [1.04, 2.60]	1.81 [1.31, 2.34]
Health expenditure per capita (USD)	90 [47, 219]	26 [15, 87]	39 [26, 91]	85 [45, 169]	101 [64, 219]	110 [61, 247]	118 [68, 288]	157 [76, 364]
**UHC service coverage index (N=126)**	54 [44, 66]	36 [25, 44]	38 [33, 49]	50 [42, 59]	57 [47, 68]	60 [54, 73]	62 [56, 72]	61 [54, 68]
**DALYs – All causes (per 100,000) (N=126)**	35,080 [28,165, 42,158]	41,384 [34,551, 56,170]	38,429 [31,195, 48,129]	37,378 [29,022, 42,088]	35,269 [27,245, 39,999]	34,093 [26,488, 38,777]	33,153 [26,124, 37,666]	33,782 [27,482, 42,169]

Note: §The table shows the median [interquartile range (IQR)], N: 18 countries and 7 years (total 126). Abbreviations: UHC, universal health coverage; USD, United States dollar; DALYs, disability-adjusted life years; GDP, gross domestic product; NA, missing values.
